# Can acute clinical outcomes predict health-related quality of life after stroke: a one-year prospective study of stroke survivors

**DOI:** 10.1186/s12955-018-1043-3

**Published:** 2018-11-21

**Authors:** Yen Shing Yeoh, Gerald Choon-Huat Koh, Chuen Seng Tan, Kim En Lee, Tian Ming Tu, Rajinder Singh, Hui Meng Chang, Deidre A. De Silva, Yee Sien Ng, Yan Hoon Ang, Philip Yap, Effie Chew, Reshma Aziz Merchant, Tseng Tsai Yeo, Ning Chou, N. Venketasubramanian, Sherry H. Young, Helen Hoenig, David Bruce Matchar, Nan Luo

**Affiliations:** 1 Saw Swee Hock School of Public Health, National University of Singapore, Tahir Foundation Building, Block MD1, 12 Science Drive 2, Singapore, 117549 Singapore; 2 Farrer Park Hospital, 1 Farrer Park Station Road, #10-08 Connexion, Singapore, Singapore; 3 National Neuroscience Institute, 11 Jalan Tan Tock Seng, Singapore, Singapore; 4 Department of Neurology, Singapore General Hospital campus, National Neuroscience Institute, 20 College Road, Singapore, Singapore; 5 Department of Rehabilitation Medicine, Singapore General Hospital, 20 College Road, Singapore, Singapore; 6 Geriatric Medicine, Khoo Teck Puat Hospital, 90 Yishun Central, Singapore, Singapore; 7 Department of Rehabilitation Medicine, National University Hospital, 5 Lower Kent Ridge Road, Singapore, Singapore; 8 Department of Medicine, Yong Loo Lin School of Medicine, National University of Singapore, Singapore, Singapore; 9 Department of Neurosurgery, National University Hospital, 5 Lower Kent Ridge Road, Singapore, Singapore; 10 Raffles Neuroscience Centre, Raffles Hospital, 585 North Bridge Road, Singapore, Singapore; 11 Department of Rehabilitation Medicine, Changi General Hospital, 2 Simei Street 3, Singapore, Singapore; 12 Veterans Affairs Medical Center, 508 Fulton St, Durham, NC USA; 13 Duke University Medical Center, Duke Box, Durham, NC 3003 USA; 14 Duke-NUS Medical School, 8 College Road, Singapore, Singapore; 15 Center for Clinical Health Policy Research, Duke University Medical Center, First Union Tower, 2200 W Main St, Suite, Durham, NC 230 USA

**Keywords:** Health-related quality of life, Stroke, Clinical outcome measures, EQ-5D-3 L, Shah-modified Barthel index, Modified Rankin scale, National Institute of health stroke scale, Mini-mental state examination, Frontal assessment battery

## Abstract

**Background:**

Health-related quality of life (HRQoL) is a key metric to understand the impact of stroke from patients’ perspective. Yet HRQoL is not readily measured in clinical practice. This study aims to investigate the extent to which clinical outcomes during admission predict HRQoL at 3 months and 1 year post-stroke.

**Methods:**

Stroke patients admitted to five tertiary hospitals in Singapore were assessed with Shah-modified Barthel Index (Shah-mBI), National Institute of Health Stroke Scale (NIHSS), Modified Rankin Scale (mRS), Mini-Mental State Examination (MMSE), and Frontal Assessment Battery (FAB) before discharge, and the EQ-5D questionnaire at 3 months and 12 months post-stroke. Association of clinical measures with the EQ index at both time points was examined using multiple linear regression models. Forward stepwise selection was applied and consistently significant clinical measures were analyzed for their association with individual dimensions of EQ-5D in multiple logistic regressions.

**Results:**

All five clinical measures at baseline were significant predictors of the EQ index at 3 months and 12 months, except that MMSE was not significantly associated with the EQ index at 12 months. NIHSS (3-month standardized β = − 0.111; 12-month standardized β = − 0.109) and mRS (3-month standardized β = − 0.122; 12-month standardized β = − 0.080) were shown to have a larger effect size than other measures. The contribution of NIHSS and mRS as significant predictors of HRQoL was mostly explained by their association with the mobility, self-care, and usual activities dimensions of EQ-5D.

**Conclusions:**

HRQoL at 3 months and 12 months post-stroke can be predicted by clinical outcomes in the acute phase. NIHSS and mRS are better predictors than BI, MMSE, and FAB.

## Background

Stroke has considerable adverse physical and psychological impact on stroke survivors [1]. Alteration in functional ability, mood disorders, cognitive impairment and decreased social interaction are commonly seen in post-stroke survivors [2]. A multitude of assessment tools are used by healthcare professionals to evaluate these changes. Despite the rather straightforward interpretations, the debilitating effects of stroke may not be fully captured solely with these tools [3]. In line with patient-centered healthcare, there is growing consensus that health-related quality of life (HRQoL) is a key metric with which to understand the impact of disease from patients’ perspective [4, 5].

Many studies have investigated predictors or determinants of quality of life after stroke, and some clinical predictors were identified [5–9]. Comparison between studies was difficult, partly due to substantial methodological differences across studies and the inherent heterogeneity of stroke severity [6, 8, 10]. In most studies, only univariate analyses have been performed [6]. Furthermore, many prior studies have been cross-sectional which assessed the association between factors and HRQoL at the same time point [6]. The use of different HRQoL instruments and different timing at which HRQoL was measured have also contributed to the inconclusive findings [9, 10].

Despite the growing recognition of HRQoL in management of stroke survivors, HRQoL is not measured routinely in clinical practice. Little is known whether the clinical measures were reflective of quality of life after stroke. A previous cross-sectional study which examined the relationship between HRQoL and clinical measures at 3 months after stroke concluded that modified rankin scale (mRS), a commonly used clinical measure for patients’ disability level in stroke trial, aligned closely with patients’ quality of life [11]. On the other hand, Katzan et al. showed that mRS demonstrated a significant ceiling effect and failed to delineate patients’ perceived health status [12].

In the present study, we investigated multiple commonly used clinical measures for their predictive value for HRQoL at 3 months and 1 year post-stroke, respectively. We hypothesize that some of these clinical measures may be a useful predictor of post-stroke HRQoL.

## Methods

### Samplings and procedure

Consecutive acute stroke patients admitted to inpatient stroke units of five public tertiary hospitals in Singapore -- Changi General Hospital, Khoo Teck Puat Hospital, National University Hospital, National Neuroscience Institutes at Tan Tock Seng Hospital and Singapore General Hospital between November 2011 to October 2013 were recruited. The eligible criteria were: 1) Singaporean or permanent resident; 2) aged 40 years or above;3) a confirmed clinical diagnosis of stroke by clinician and/or supported by neuroimaging.

A battery of clinical measures and a HRQoL measure were administered by a trained interviewer during the hospitalization period. For the HRQoL measure, in the case where patients were unable to respond to the questions by themselves, proxy responses were obtained from their caregivers, if available. Patients were followed-up at 3 and 12 months post-stroke in their homes. A mail reminder was sent out in advance with phone calls or text messages made one day before the visits. All other data was obtained from medical records or face-to-face interviews with patients or their caregivers. A set of data collection guidelines was created for standardization of data collection and field work procedures among different tertiary hospitals.

Full disclosure was provided to the subjects before obtaining informed consent. The study was approved by SingHealth Centralized Institutional Review Board and National Healthcare Group Domain Specific Review Board.

### Instruments

Post-stroke HRQoL was assessed with the European Quality of Life Five Dimensions - Three Levels (EQ-5D-3 L) [13]. It has been validated in many countries and shown to be a credible instrument in measuring post-stroke HRQoL [1, 14, 15]. It consists of five dimensions that assess mobility, self-care, usual activities, pain/discomfort, and anxiety/depression. Each dimension is evaluated using three levels of severity (no problems, some or moderate problems, extreme problems). The resulting EQ index anchored by 0 (dead) and 1 (full health) was then calculated using the value set for Singapore [16]. In the current study, HRQoL at baseline reflected patient’s health status on a typical day before stroke, while health status at follow-up time points reflected health status on the day of interview itself.

Clinical measures assessed at baseline in this study included Shah-modified BI (Shah-mBI) [17] on a 0–100 scale which measures ability of stroke patients to perform activities of daily living; mRS [18], a single item scale with 7 grades which assesses disability of patients; National Institute of Health Stroke Scale (NIHSS) [19] which provides a score of stroke-related neurological deficits ranging between 0 and 42; Mini-Mental State Examination (MMSE) [20] which evaluates cognitive impairment with a score ranging between 0 and30; and Frontal Assessment Battery (FAB) [21] which measures executive dysfunctions associated with functional impairment in stroke on a 0–18 scale.

### Statistical analysis

Means and standard deviations (continuous variables) and counts and percentages (categorical variables) were used for descriptive statistics. All clinical measures were treated as continuous scales. The clinical measures were calculated by employing a “half-item rule” [22, 23]. This rule allowed the calculation of scale scores for patients who had missing data for less than half of the items constituting a scale. Based on this rule, the missing data is imputed with the average score of completed items in the same scale.

Simple linear regressions were first performed to examine the association between the EQ index at 3 months and each of the clinical measure at baseline (Model 1). The association of each clinical measure with the EQ index was adjusted for socio-demographic and health-related variables by using a multiple liner regression model (Model 2). Age, gender, ethnicity, marital status, religion, presence of caregiver, hospital sites, ward class, baseline survey mode, 3-month or 12-month survey mode, stroke subtype, stroke episode, Charlson Comorbidities Index (CCI), baseline (pre-stroke) EQ index value, Center of Epidemiologic Studies Depression Scale were examined individually for their effects on the EQ index using simple linear regression models, and those socio-demographic and health-related variables with *p*-value < 0.1 were included in Model 2. Lastly, a parsimonious model for EQ index was obtained via forward stepwise selection (Model 3) by using a list of potential predictors consisted of all five clinical measures and the socio-demographic and health-related variables included in Model 2. The threshold *p*-values for the variable to enter the model and remove from the model were 0.05 and 0.1, respectively. Standardized regression coefficients of the clinical measures were reported to compare their relative effect sizes in predicting the EQ index. To account for multiple testing on five clinical measures with Bonferroni correction, *p* < 0.01 (=0.05/5) was considered significant.

To understand the attributes of individual EQ-5D dimensions for the effect of clinical measures, multiple logistic regressions were employed to further analyze the association between clinical measures shown to be consistently associated with the EQ index in Model 3 with individual EQ-5D dimensions. Binary logistic regression analyses were employed by dichotomizing each three-level EQ-5D dimension into “no problem (as reference point)” and “some or severe problem” [24]. Socio-demographic and health-related variables were adjusted in the multiple logistic models the same method as performed in the linear regression models. Standardized odds ratios were reported to estimate the relative effect size of the clinical measures in predicting problems in each EQ dimension. To account for multiple testing for each clinical measure on the five EQ-5D dimensions with Bonferroni correction, p < 0.01 (=0.05/5) was considered significant. All analyses were repeated for the EQ index at 12 months and performed using Stata version 11.0 [25].

## Results

**Fig. 1:**
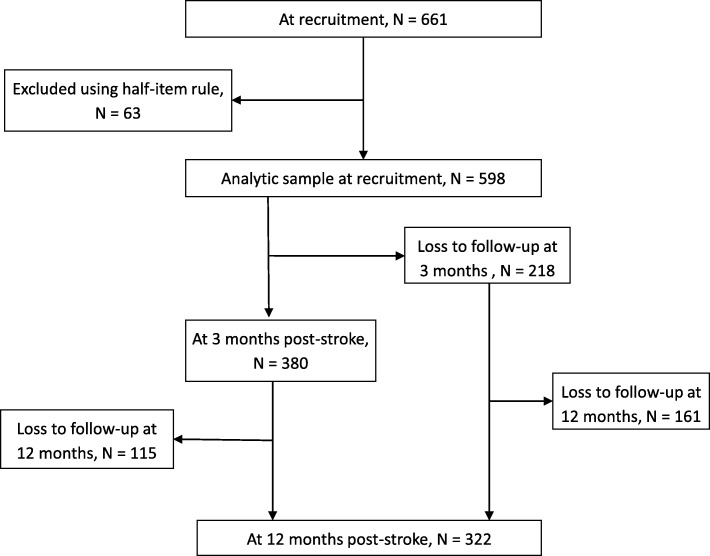
ᅟ

Of the total 661 patients recruited, 63 patients were excluded after applying “half-item rule” [22, 23]. Among the 598 patients, 63.5% (*N* = 380) and 53.8% (*N* = 322) were followed up at 3 and 12 months, respectively, and included in the analysis (Fig. 1). Among those who were lost to followed-up, five were due to being deceased at 3 months post-stroke.

At baseline, the study sample mainly consisted of patients with ischemic stroke (89%) and about 81% of patients were having their first episode of stroke. The majority of the patients were male (66%), age 60 years and above (mean age: 62.2 years) and ethnic Chinese (68.2%). Most of the patients were married (69.6%) and half were having spouse as their primary caregiver at baseline. A comparison of baseline characteristics between the follow-up and loss to follow-up (LTFU) at 3 months and 12 months post-stroke are showed in Table 1. There was no significant difference in the baseline characteristics between those who were followed-up and LTFU, except marital status.

**Table 1:** Baseline characteristics between follow-up and loss to follow-up at 3 months or 12 months

	3 months		*p* value	12 months		*p* value
	Follow-up	Loss to follow-up		Follow-up	Loss to follow-up	
	*N* = 380	*N* = 218		*N* = 322	*N* = 276	
	*N* (%)	*N* (%)		*N* (%)	*N* (%)	
Age, mean (sd)	62.3 (10.6)	62.0 (11.0)	0.758	62.1 (10.6)	62.4 (11.0)	0.718
Gender			0.731			0.273
Male	251 (66.0)	147 (67.4)		208 (64.6)	190 (68.8)	
Female	129 (34.0)	71 (32.6)		114 (35.4)	86 (31.2)	
Ethnicity			0.130			0.452
Chinese	249 (65.5)	159 (72.9)		214 (66.5)	194 (70.3)	
Malay	94 (24.7)	39 (17.9)		78 (24.2)	55 (19.9)	
Indian and others	37 (9.8)	20 (9.2)		30 (9.3)	27 (9.8)	
Marital status			0.001			0.049
Single	38 (10.0)	36 (16.5)		31 (9.6)	43 (15.6)	
Married	282 (74.2)	134 (61.5)		239 (74.2)	177 (64.1)	
Separated/ Divorced	14 (3.7)	22 (10.1)		17 (5.3)	19 (6.9)	
Widowed	46 (12.1)	26 (11.9)		35 (10.9)	37 (13.4)	
Primary caregiver			0.186			0.170
Spouse	204 (54.0)	98 (45.4)		175 (54.5)	127 (46.5)	
Child	92 (24.3)	55 (25.5)		75 (23.4)	72 (26.4)	
Sibling	19 (5.0)	19 (8.8)		21 (6.5)	17 (6.2)	
Maid/ others	21 (5.6)	15 (6.9)		20 (6.2)	16 (5.9)	
None	42 (11.1)	29 (13.4)		30 (9.4)	41 (15.0)	
Survey mode at baseline			0.721			0.766
Stroke patient	358 (94.2)	205 (94.9)		305 (94.7)	258 (94.2)	
Primary caregiver	22 (5.8)	11 (5.1)		17 (5.3)	16 (5.8)	
Subtype of stroke			0.414			0.876
Infarct (Ischemic)	341 (89.7)	190 (87.6)		287 (89.1)	244 (88.7)	
Haemorrhage/ both	39 (10.3)	27 (12.4)		35 (10.9)	31 (11.3)	
Episode of stroke			0.809			0.637
First stroke	309 (81.3)	179 (82.1)		265 (82.3)	223 (80.8)	
Recurrent stroke	71 (18.7)	39 (17.9)		57 (17.7)	53 (19.2)	
CCI, mean (sd)	5.00 (1.70)	4.85 (1.87)	0.320	4.95 (1.73)	4.95 (1.82)	0.989
EQ index value at baseline, mean (sd)	0.83 (0.26)	0.84 (0.25)	0.577	0.83 (0.27)	0.84 (0.25)	0.489
CES-D, mean (sd)	6.32 (5.48)	6.90 (5.48)	0.207	6.56 (5.41)	6.50 (5.57)	0.901
Shah-mBI, mean (sd)	71.79 (30.62)	71.75 (31.21)	0.986	70.38 (31.45)	73.40 (30.03)	0.232
NIHSS, mean (sd)	4.74 (4.57)	4.51 (4.50)	0.547	4.57 (4.42)	4.75 (4.69)	0.626
MMSE, mean (sd)	23.23 (6.26)	22.67 (6.39)	0.295	23.28 (6.21)	22.73 (6.42)	0.290
FAB, mean (sd)	13.45 (4.51)	13.65 (4.32)	0.594	13.81 (4.27)	13.19 (4.60)	0.087
mRS, mean (sd)	2.65 (1.33)	2.49 (1.41)	0.151	2.61 (1.37)	2.58 (1.36)	0.792
mRS			0.207			0.332
No symptoms at all	8 (2.1)	11 (5.0)		7 (2.2)	12 (4.4)	
No significant disability	104 (27.4)	66 (30.3)		98 (30.4)	72 (26.1)	
Slight disability	54 (14.2)	32 (14.7)		43 (13.4)	43 (15.6)	
Moderate disability	72 (18.9)	29 (13.3)		53 (16.5)	48 (17.4)	
Moderately severe disability	130 (34.2)	75 (34.4)		109 (33.8)	96 (34.8)	
Severe disability	12 (3.2)	5 (2.3)		12 (3.7)	5 (1.8)	

*sd*: standard deviation; *CCI*: Charlson Comorbidities Index; *CES-D*: Center of Epidemiologic Studies Depression Scale; *Shah-mBI*: Shah-modified Barthel Index; *NIHSS*: National Institute of Health Stroke Scale; *MMSE*: Mini-Mental State Examination; *FAB*: Frontal Battery Assessment; *mRS*: Modified Rankin Scale

Table 2 shows the association between acute clinical measures at baseline and the EQ index at 3 months post-stroke. In the simple linear regression (Model 1), all clinical measures were associated with the EQ index at 3 months after correcting for multiple testing (all *p* < 0.01). After adjustment for socio-demographic and health-related variables (Model 2), the association remained statistically significant for all clinical measures (p < 0.01). In the final model (Model 3), three of the five clinical measures entered the final model with adjustment for socio-demographic and health-related variables: NIHSS (standardized β = − 0.111), mRS (standardized β = − 0.122) and FAB (standardized β = 0.058) which remained significant after correcting for multiple testing.

**Table 2:** Associations between acute clinical measures at baseline and the EQ index at 3 months post-stroke (N = 380)

	Model 1		Model 2		Model 3	
	B^a^ (95%CI)	B^b^	B^a^ (95%CI)	B^b^	B^a^ (95%CI)	B^b^
Shah-mBI	0.007 (0.006; 0.009)	0.221ǂ	0.005 (0.004; 0.007)	0.156ǂ		
NIHSS	−0.054 (−0.063; −0.046)	−0.248ǂ	− 0.043 (− 0.053; − 0.034)	−0.198ǂ	− 0.024 (− 0.035; − 0.013)	−0.111ǂ
MMSE	0.029 (0.022; 0.036)	0.181ǂ	0.013 (0.004; 0.022)	0.084ǂ		
FAB	0.043 (0.034; 0.053)	0.194ǂ	0.027 (0.015; 0.039)	0.121ǂ	0.013 (− 0.003; − 0.023)	0.058ǂ
mRS	−0.179 (− 0.209; − 0.149)	−0.239ǂ	− 0.130 (− 0.163; − 0.097)	−0.174ǂ	− 0.091 (− 0.126; − 0.057)	−0.122ǂ

Model 1: separate simple linear regression for each clinical measure; Model 2: separate multiple linear regression for each clinical measure with adjustment for age, gender, marital status, hospital sites, baseline survey mode, 3-month survey mode, stroke subtype, stroke episode, Charlson Comorbidities Index, baseline EQ index value, Center of Epidemiologic Studies Depression Scale; Model 3: final model obtained from forward stepwise selection method where clinical measures are in the same common model with adjustment for 3-month survey mode, baseline EQ index value and Center of Epidemiologic Studies Depression Scale

B^a^: unstandardized coefficient; B^b^: standardized coefficient; *Shah-mBI*: Shah-modified Barthel Index; *NIHSS*: National Institute of Health Stroke Scale; *MMSE*: Mini-Mental State Examination; *FAB*: Frontal Assessments Battery; *mRS*: modified Rankin Scale

ǂ *p* value < 0.01

The association between acute clinical measures at baseline and the EQ index at 12 months post-stroke is shown in Table 3. In both Model 1 and Model 2, all clinical measures were significantly associated with the EQ index at 12 months post-stroke, except MMSE was not significant after adjusting for socio-demographic and health-related variables and Bonferroni correction. In Model 3, only NIHSS (standardized β = − 0.109) and mRS (standardized β = − 0.080) were significant and retained.

**Table 3:** Associations between acute clinical measures at baseline and the EQ index at 12 months post-stroke (N = 322)

	Model 1		Model 2		Model 3	
	B^a^ (95%CI)	B^b^	B^a^ (95%CI)	B^b^	B^a^ (95%CI)	B^b^
Shah-mBI	0.005 (0.004; 0.007)	0.171ǂ	0.003 (0.002; 0.005)	0.107ǂ		
NIHSS	−0.045 (−0.055; − 0.036)	−0.197ǂ	− 0.034 (− 0.043; − 0.025)	−0.147ǂ	− 0.025 (− 0.035; − 0.015)	−0.109ǂ
MMSE	0.026 (0.019; 0.033)	0.161ǂ	0.008 (0.001; 0.016)	0.053		
FAB	0.044 (0.034; 0.053)	0.186ǂ	0.019 (0.007; 0.031)	0.081ǂ		
mRS	−0.136 (−0.167; − 0.106)	−0.187ǂ	− 0.102 (− 0.131; − 0.073)	−0.140ǂ	− 0.058 (− 0.091; − 0.026)	−0.080ǂ

Model 1: separate simple linear regression for each clinical measure; Model 2: separate multiple linear regression for each clinical measure with adjustment for age, gender, marital status, hospital sites, baseline survey mode, 12-month survey mode, stroke subtype, stroke episode, Charlson Comorbidities Index, baseline EQ index value, Center of Epidemiologic Studies Depression Scale; Model 3: Final model obtained from forward stepwise selection method where clinical measures are in the same common model with adjustment for 12-month survey mode, baseline EQ index value, age, hospital sites and stroke episode

B^a^: unstandardized coefficient; B^b^: standardized coefficient; *Shah-mBI*: Shah-modified Barthel Index; *NIHSS*: National Institute of Health Stroke Scale; *MMSE*: Mini-Mental State Examination; *FAB*: Frontal Assessments Battery; *mRS*: modified Rankin Scale

ǂ *p* value < 0.01

Association between NIHSS, mRS or FAB with each of the EQ dimension is shown in Table 4. At 3 months post-stroke, mRS was significantly associated with all the EQ dimensions. Similar findings were observed in NIHSS except that NIHSS was not significant in anxiety/depression dimension. FAB was significantly associated with mobility, self-care and usual activities but not pain/discomfort and anxiety/depression. For both NIHSS and mRS, the effect sizes were larger in the first three dimensions as compared to the last two. At 12 months post-stroke, both NIHSS and mRS were significant independent predictors for dimension of mobility, self-care and usual activities after Bonferroni correction.

**Table 4:** Association between NIHSS or mRS or FAB and individual EQ-5D dimensions

	3 months			12 months		
	Unstandardized OR (95% CI)	Standardized OR	*p* value	Unstandardized OR (95% CI)	Standardized OR	*p* value
Mobility						
NIHSS	1.27 (1.18; 1.38)	3.03	< 0.001	1.16 (1.08; 1.25)	1.92	< 0.001
mRS	2.10 (1.65; 2.66)	2.68	< 0.001	1.61 (1.28; 2.02)	1.92	< 0.001
FAB	0.87 (0.81;0.94)	0.54	< 0.001			
Self-care						
NIHSS	1.35 (1.23; 1.47)	3.91	< 0.001	1.34 (1.22; 1.48)	3.67	< 0.001
mRS	2.82 (2.02; 3.94)	3.99	< 0.001	2.52 (1.80; 3.55)	3.57	< 0.001
FAB	0.88 (0.82;0.95)	0.57	0.001			
Usual Activities						
NIHSS	1.30 (1.20; 1.41)	3.33	< 0.001	1.28 (1.18; 1.40)	2.99	< 0.001
mRS	2.43 (1.88; 3.14)	3.27	< 0.001	2.59 (1.93; 3.48)	3.69	< 0.001
FAB	0.88 (0.82;0.94)	0.56	< 0.001			
Pain/Discomfort						
NIHSS	1.09 (1.03; 1.14)	1.45	0.003	1.06 (1.00; 1.13)	1.32	0.047
mRS	1.28 (1.09; 1.51)	1.39	0.004	1.30 (1.05; 1.62)	1.44	0.017
FAB	0.97 (0.92;1.02)	0.86	0.186			
Anxiety/Depression						
NIHSS	1.04 (1.00; 1.09)	1.22	0.072	1.03 (0.96; 1.11)	1.16	0.351
mRS	1.27 (1.07; 1.50)	1.37	0.006	1.26 (1.01; 1.59)	1.38	0.043
FAB	0.97 (0.93;1.02)	0.89	0.275			

Models were adjusted for the following variables where *p* < 0.1 in univariate analysis: age, gender, ethnicity, marital status, religion, primary caregiver, hospital sites, ward class, baseline survey mode, 3-month survey mode, stroke subtype, stroke episode, Charlson Comorbidities Index, baseline EQ index value, Center of Epidemiologic Studies Depression Scale

*OR*: odds ratio; *NIHSS*: National Institute of Health Stroke Scale; *mRS*: modified Rankin Scale

## Discussion

We observed that among the clinical measures commonly performed in acute phase stroke survivors, NIHSS and mRS were consistently independent predictors of HRQoL at 3 and 12 months with comparable effect sizes. When looking into the effect of NIHSS and mRS on each EQ dimension, it was not surprising that NIHSS and mRS were better predictors of health problems in the dimensions of mobility, self-care and usual activities than pain/discomfort and anxiety/depression. Our results that baseline NIHSS score was an independent predictor of HRQoL not only confirmed the association between baseline NIHSS score and HRQoL among stroke survivors observed in a few cross-sectional studies [11, 26], it is also consistent with the findings from Christensen et al. [6], Fischer et al. [7], and Sturm et al. [26], which demonstrated the predictability of baseline NIHSS score for HRQoL ranging from 3 months up to 2 years post-stroke.

The National Institute of Neurological Disorder and Stroke Common Data Elements (NINDS CDE) and the European Stroke Organization Outcome Working Group have recommended mRS as a robust primary outcome measure to be used in acute stroke trials. In a cross-sectional study by Ali et al., mRS, which was able to capture more information on quality of life than either NIHSS or BI, was concluded as a useful indicator of patient’s overall HRQoL at 3 months post-stroke [11]. Fischer et al. found that patients with a high mRS score and a low BI score in both scales had more impaired quality of life [7]. In our study, mRS was prospectively assessed and found to be independently associated with both HRQoL at 3 months and 12 months post-stroke with relatively larger effect size compared to other measures. On the other hand, BI which was significantly associated with HRQoL as demonstrated in some cross-sectional studies [7, 11, 26], was found to be insignificant for HRQoL at 1 year and 2.5 years post-stroke in a longitudinal study [8]. In our study, although BI was independently associated with HRQoL at 3 months and 12 months, its effect size was smaller when compared with other measures, in particular the NIHSS and mRS, in the final model. This may be attributed to the ceiling and floor effects and the lack of responsiveness to deficits through the range of expected outcomes [27].

Our study had some limitations. As with most prospective cohort studies, LTFU is one of the largest threats in the study design. Several measures such as home visiting, reminder calls and text messaging, and incentives had been implemented to mitigate the LTFU bias, despite the LTFU rate of about 36 and 46% at 3 and 12 months, respectively. However, there was little difference in the characteristic of patients who were followed-up and who were not. Thus the data was generalizable to the whole study cohort. Proxy responses were used when patients were unable to answer the questions by themselves during the immediate days after stroke. Although studies have shown proxies systematically rated impairments worse than patients themselves [28–30], Delcourt et al. found that the predictor factors for HRQoL were similar between proxy and patient [31]. In addition, up to 80% of patients in our study evaluated their own HRQoL at baseline and responses from proxies were minor, thereby providing robust data from the patients’ perspective. In our study, patients with transient ischemic attack who did not have residual post-stroke deficits and patients who were terminally-ill were not included. This may fail to reflect the wide range of stroke patients with varying severities and disabilities. As shown by the mean mRS score, our patient cohort mostly suffered from mild to moderate deficits. Our findings may therefore have limited generalizability to stroke patients with moderate to severe deficits.

Our study had several strengths. In our study, patients were recruited from stroke units in all government restructured hospitals in Singapore at time of study. More than 90% of Singaporeans with acute stroke are admitted to one of the five restructured hospitals and less commonly to private general hospital [32]. The sampling frame is hence likely sufficient for representativeness on national level. In our models, patients’ pre-stroke health status which could potentially affect the subsequent HRQoL measure was adjusted in all analyses. To our knowledge, only one past study has considered pre-stroke health status in study of HRQoL changes in patients after stroke [33].

The chosen clinical measures were commonly used in acute stroke survivors [34–36]. The chosen HRQoL instrument, the EQ-5D, has been shown to be a valid measure of post-stroke HRQoL [14] and was recommended as measures of participation for data collection by the NINDS CDE project [11]. Apart from self-reporting, its usage with proxy respondents has also been evaluated and confirmed [29, 30]. For administration of clinical and HRQoL measures, all research staff and interviewers underwent standardized and proper training and certification, thereby minimizing inter-rater variability.

## Conclusions

There was significant association between acute phase clinical measures and HRQoL of Singaporean stroke survivors at both 3 and 12 months post-stroke. NIHSS and mRS were independent predictors of post-stroke HRQoL with larger effect sizes than other clinical measures, suggesting their usefulness as indicators for patients’ quality of life after stroke. Future research is warranted to investigate whether these findings may be generalizable to survivors with severe post-stroke deficits.

## Abbreviations

CCI: Charlson Comorbidities Index
EQ-5D-3 L: The European Quality of Life Five Dimensions - Three Levels
FAB: Frontal Assessment Battery
HRQoL: Health-related quality of life
LTFU: Loss to follow-up
MMSE: Mini-Mental State Examination
mRS: Modified Rankin Scale
NIHSS: National Institute of Health Stroke Scale
NINDS CDE: The National Institute of Neurological Disorder and Stroke Common Data Elements
Shah-mBI: Shah-modified Barthel Index
